# Poly (ADP‐ribose) polymerase 1 inhibition prevents neurodegeneration and promotes α‐synuclein degradation via transcription factor EB‐dependent autophagy in mutant α‐synucleinA53T model of Parkinson's disease

**DOI:** 10.1111/acel.13163

**Published:** 2020-05-31

**Authors:** Kanmin Mao, Jialong Chen, Honglin Yu, Huihui Li, Yixian Ren, Xian Wu, Yue Wen, Fei Zou, Wenjun Li

**Affiliations:** ^1^ Department of Occupational Health and Occupational Medicine Guangdong Provincial Key Laboratory of Tropical Disease Research School of Public Health Southern Medical University Guangzhou China

**Keywords:** autophagy, Parkinson's disease, PARP1, SIRT1, TFEB

## Abstract

Poly (ADP‐ribose) polymerase 1 (PARP1) is a master regulator of diverse biological processes such as DNA repair, oxidative stress, and apoptosis. PARP1 can be activated by aggregated α‐synuclein, and this process in turn exacerbates toxicity of α‐synuclein. This circle is closely linked to the evolution of Parkinson's disease (PD) that characterized by progressive neurodegeneration and motor deficits. Here, we reported the PARP1, as a novel upstream molecular of transcription factor EB (TFEB), participates in regulation of autophagy in α‐synuclein aggregated cells and mice. PARP1 inhibition not only enhances the nuclear transcription of TFEB via SIRT1 mediated down‐regulation of mTOR signaling but also reduces nuclear export of TFEB by attenuating the TFEB‐CRM1 interaction. Our results revealed that PARP1 inhibition lessened the accumulation of α‐synuclein in PD models. Also, oral administration of PARP1 inhibitor Veliparib prevented neurodegeneration and improved motor ability in α‐synucleinA53T transgenic mice. These findings identify that PARP1 signaling pathway regulates TFEB‐mediated autophagy, pointing to potential therapeutic strategy of PD via enhancing protein degradation systems.

## Graphical abstract

α‐synuclein aggregated induced DNA damaged and Poly (ADP‐ribose) polymerase 1 (PARP1) activation, which down‐regulated transcription factor EB (TFEB) nuclear translocation via TFEB PARylation or TFEB phosphorylations regulated by PAR and SIRT1‐mTOR pathways, separately. Inhibition of PARP1 promotes TFEB‐mediated autophagy and then increases the degradation of α‐synuclein, providing a potential therapeutic strategy of Parkinson’s disease.

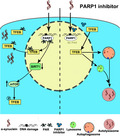

## 1 INTRODUCTION

Parkinson's disease (PD) is an age‐related neurodegenerative disease characterized by uncoordinated movement, dementia, alexithymia, and dyssomnia (Ferreri, Agbokou, & Gauthier, 2006). The typical pathological features of PD are progressive loss of dopamine (DA) neurons in substantia nigra and formation of Lewy bodies. Autosomal dominant PD is mainly caused by mutation of SNCA gene as A53T, E57K, and A30P, which encodes misfolded α‐synuclein protein as the major component of Lewy body in PD patients’ brain. It has been proved that α‐synuclein accumulation causes neuritic and synaptic degeneration preceding neuronal death (Peelaerts & Baekelandt, 2016), but the way of blocking aggregation of pathological α‐synuclein is poorly understood.

In general, α‐synuclein degradation depends on ubiquitin‐proteasome system and autophagy–lysosomal pathway. Defects in these proteolytic systems cause aggregation of α‐synuclein, which contributes to the pathogenesis of PD (Chen et al., 2018; Cuervo & Wong, 2014). Thus, the study on the molecular mechanisms of autophagy dysfunction may help us to better understand the etiology of PD. Transcription factor EB (TFEB) is a main regulator of autophagy, which positively regulates autophagosome formation and fusion of autophagosome‐lysosome (Settembre, Fraldi, Medina, & Ballabio, 2013). The activity of TFEB is closely related to its cellular localization regulated by mTOR, ERK, and other molecules. Here, we propose a new regulator of TFEB to regulate its subcellular localization and then participate in autophagy–lysosomal pathway.

Poly (ADP‐ribose) polymerase 1 (PARP1) is an enzyme activated by DNA damage. Using NAD^+^ as substrate, PARP1 promotes the formation of poly (ADP‐ribose) polymers (PAR) on receptor proteins (Ke et al., 2017), regulating various physiological processes such as maintain of genomic stability. Previous reports showed that activated PARP1 can induce mitochondrial dysfunction and modify α‐synuclein into more toxic strains, accelerating the pathological progress of PD (Kam et al., 2018). In addition, PARP1 also interacts with AMPK (Rodriguez‐Vargas et al., 2016). AMPK is a key molecule in bioenergy metabolism and participates in the activation of autophagy during cell starvation. Of note, Abby L. Olsen proposed that PARP1 inhibitor can be used for the treatment of PD, but its mechanism needs to be further explored (Olsen & Feany, 2019). Therefore, we investigated the PARP1 and its relationship to autophagy in PD models.

Here, we provide direct evidences that PARP1 inhibitor reduces aggregated α‐synuclein on consequence of TFEB‐mediated autophagy. Results present that attenuated neurological deficits and strengthen motor ability after inhibition of PARP1 in vivo models of PD. These results suggest that PARP1 inhibitor may be a potential therapeutic strategy for PD via enhancing α‐synuclein degradation.

## 2 RESULTS

### 2.1 PARP1/PAR pathway is activated in α‐synucleinA53T model of PD

α‐synuclein is the major component of Lewy body, which is implicated in the pathogenesis of PD. Therefore, we investigated the effects of α‐synucleinA53T over‐expressed on C57 mice (α‐synucleinA53T transgenic mice) and SN4741 cells (α‐synucleinA53T SN4741 cells). Compared with the control group, these mice and cells expressed highly accumulated α‐synuclein (Figure S1a,b). Moreover, tail DNA isolated from mice was detected by PCR (Figure S1c).

**Figure 1:**
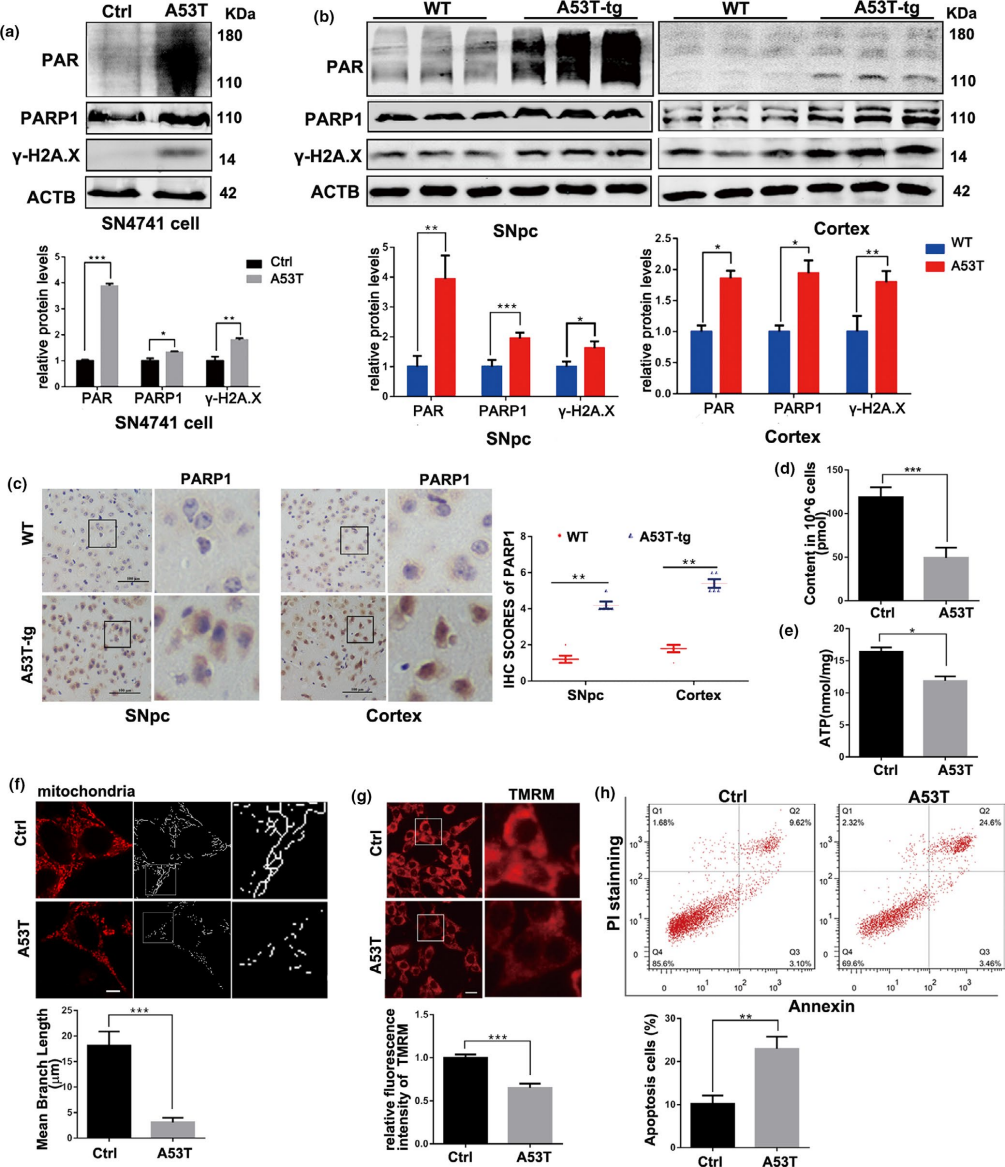
PARP1/PAR pathway is activated in overexpressed α‐synucleinA53T models of Parkinson's disease. (a) SN4741 cells were transfected with Adenovirus‐Ctrl/A53T for 36 hr. Representative immunoblots and quantification of the levels of PAR, PARP1, and γ‐H2A.X. Means ± *SEM*, *n* = 3. (b) Representative immunoblots and quantification of the levels of PAR, PARP1 and γ‐H2A.X accumulation in SNpc and Cortex section of wild‐type (WT) or α‐synuclein^A53T^‐tg mice. Means ± *SEM*, *n* = 3. (c) Immunohistochemistry (IHC) staining of PARP1 in SNpc and Cortex tissue of WT and α‐synuclein^A53T^‐tg mice. Statistical analysis of the scores of PARP1 staining also shown in c. Scale bars, 100 μm. (d,e) NAD^+^ (d) and ATP (e) levels inSN4741 cells measured by spectrophotometer. (f) Representative images of mitochondrial tracker (red) staining from SN4741 cells transfected with Adenovirus‐Ctrl/A53T, and quantification of mean branch length of Mitochondrial fragments. Means ± *SEM*, *n* = 20. Scale bars, 5 μm. (g) Representative images of TMRM (red) staining and quantification of fluorescence intensity from SN4741 cells. Means ± *SEM*, *n* = 20. Scale bars, 15 μm. (h) Apoptosis rates were measured by fluorescence‐activated cell sorting analysis. *n* = 3 (the statistical significantly was analyzed by unpaired Student's *t* test, *p < .05, **p < .01 and ***p < .001). PARP1, Poly (ADP‐ribose) polymerase 1; TMRM, tetramethylrhodamine methyl ester

Immunoblotting and immunohistochemistry (IHC) were used to detect the effects of α‐synuclein on PARP1 and PAR. The expression of PARP1 and PAR were up‐regulated in the α‐synucleinA53T SN4741 cells (Figure 1a) and the brain tissue of α‐synucleinA53T transgenic mice (Figure 1b,c). The levels of DNA damage were detected by Comet assay (Chen et al., 2011; Figure S1d) and γ‐H2A.X formation (Figure 1a,b). Importantly, results indicated that accumulation of α‐synuclein promoted the activation of PARP1 as well as DNA damage.

Previous studies have shown that the process of activating PARP1 to synthesize PAR depletes intracellular NAD^+^, resulting in mitochondrial damage (Lehmann, Costa, Celardo, Loh, & Martins, 2016). In addition, a new way of programmed neuronal death, parthanatos, has been found in Alzheimer's disease and PD (Wang et al., 2011). The death pattern is based on DNA damage and PAR formation. To test whether accumulation of α‐synuclein has toxic effects on SN4741 cells by activating PARP1, a bound of biochemical analyses were performed. Figure 1d,e showed that α‐synucleinA53T decreased the contents of NAD^+^ and ATP. Also, the increased mitochondrial fragmentation (Figure 1f) and declined mitochondrial membrane potential (MMP; Figure 1g) were measured. Moreover, the apoptosis rate of α‐synucleinA53T SN4741 cells also increased (Figure 1h). In short, the accumulation of α‐synuclein not only causes mitochondrial damage but also activates PARP1 to kill cell via a parthanatos way.

### 2.2 α‐synucleinA53T elicits autophagy dysfunction via mTOR‐TFEB pathway

**Figure 2:**
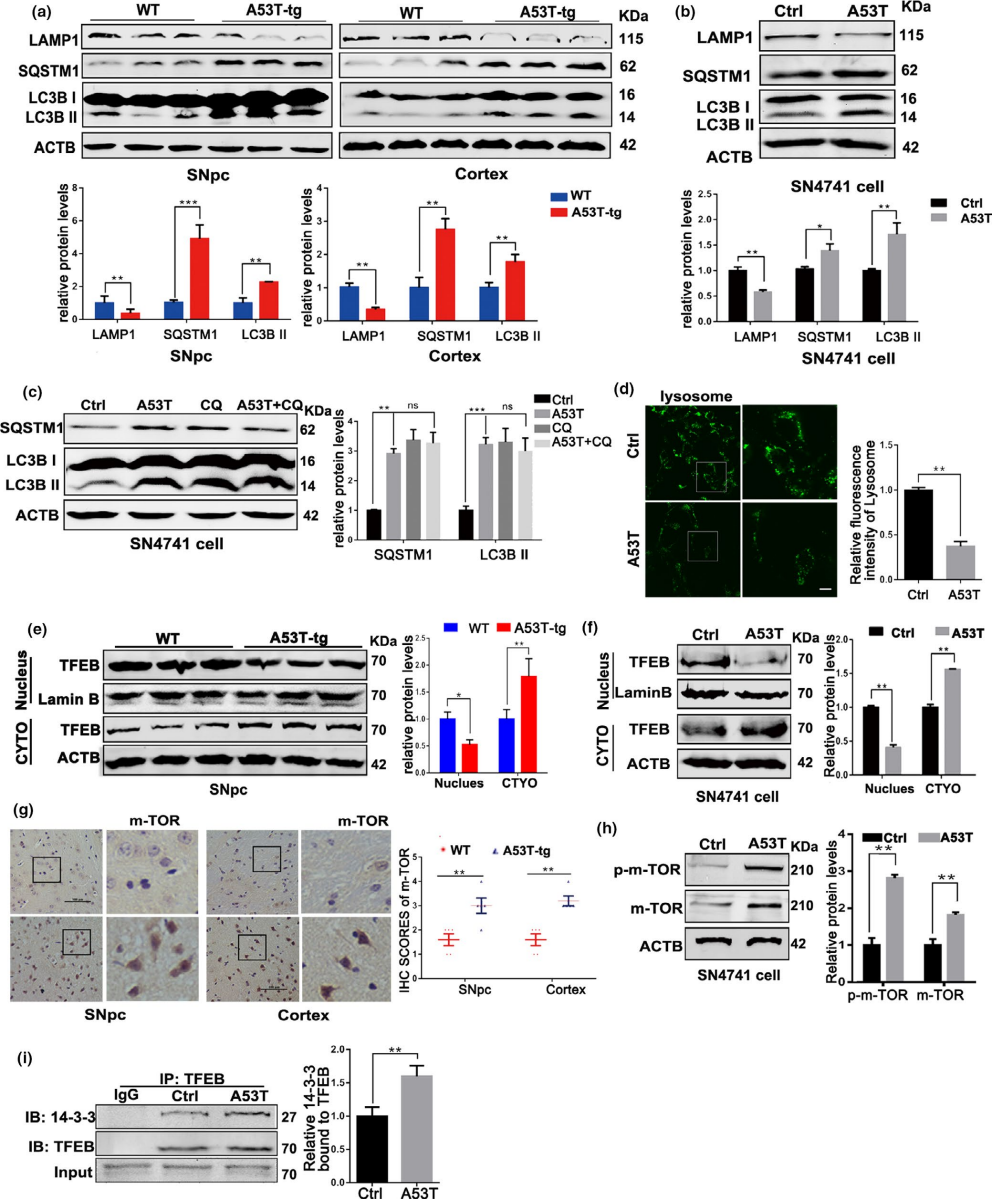
Mutant α‐synucleinA53T elicits autophagy dysfunction via mTOR‐TFEB pathway. (a) Representative immunoblots and quantification of the levels of LAMP1, SQSTM1, and LC3B II in wild‐type (WT) or α‐synucleinA53T‐tg mice. Mean ± *SEM*, *n* = 3. (b) Representative immunoblots and quantification of the levels of LAMP1, SQSTM1, and LC3B II in SN47841 cells transfected with Adenovirus‐Ctrl/A53T. Mean ± *SEM*, *n* = 3. (c) Representative immunoblots and quantification of SQSTM1 and LC3B II levels in SN4741 cells pre‐transfected with Adenovirus‐A53T and further treated with CQ (10 μM, 4 hr). Means ± *SEM*, *n* = 3. (d) Representative images and quantification of Lysosomes (green) in SN4741 cells. Scale bars, 10 μm. (e,f) Levels of TFEB from nuclear fractions, cytoplasmic fractions and total cell lysates in SNpc of mice (e) and SN4741 cell (f) were measured and analyzed. Means ± *SEM*, *n* = 3. (g) Representative mTOR staining of SNpc and Cortex tissue of WT and α‐synuclein^A53T^‐tg mice. Statistical analysis of the scores of mTOR staining shown in right. Scale bars, 100 μm. (h) Representative immunoblots and quantification of the levels of p‐mTOR and mTOR in SN4741 cells. Mean ± *SEM*, *n* = 3. (i) SN4741 cells were transfected with Adenovirus‐Ctrl/A53T. Then, the interaction between 14‐3‐3 and TFEB was measured in TFEB Immunoprecipitates. lgG worked as an immunological control. Means ± *SEM*, *n* = 3 (the statistical significantly was analyzed by unpaired Student's *t* test, *p < .05, **p < .01 and ***p < .001). TFEB, transcription factor EB

In order to assess the effect of α‐synuclein accumulation on autophagy, the levels of autophagy‐associated proteins were detected. As shown in Figure 2a,b, aggregation of α‐synucleinA53T increased the levels of SQSTM1 and LC3B II as well as decreased lysosomal marker LAMP1 both in SN4741 cell and mice, suggesting the blocked autophagy flux. Next, we treated α‐synucleinA53T SN4741 cell with chloroquine (CQ, 10 μM, 4 hr), the well‐defined lysosomal inhibitor, and lysosome dysfunction was confirmed by unchanging level of LC3B II and SQSTM1(Figure 2c). Furthermore, cellular lysosomes, dyed with LysoTracker Green, were reduced significantly in α‐synucleinA53T SN4741 cells (Figure 2d). Thus, accumulation of pathological α‐synuclein blocked autophagy flux via diminishing the number of functional lysosomes.

Since TFEB is a master regulator of lysosomal biogenesis and autophagy, we speculated whether TFEB inactivation is involved in the autophagy dysfunction caused by α‐synuclein aggregation. Immunoblotting and Immunofluorescence found that aggregation of α‐synuclein not only suppressed TFEB nuclear translocation (Figure 2e,f), but also activated mTOR (Figure 2g,h), the upstream of TFEB. Phosphorylation of TFEB at serine 211 by mTOR can promote TFEB binding to 14‐3‐3, thus preventing TFEB nuclear translocation (Ren et al., 2018). We detected the level of the interaction of 14‐3‐3/TFEB. The increasing interaction of 14‐3‐3/TFEB was confirmed in α‐synucleinA53T SN4741 cells (Figure 2i). In addition, inhibition of mTOR or over‐expression of TFEB could effectively diminish the levels of SQSTM1 and LC3BII in α‐synucleinA53T SN4741 cells (Figure S1e). These results suggested that accumulation of pathological α‐synuclein contributes to the autophagy dysfunction through TFEB‐lysosome pathway.

### 2.3 PARP1 inhibitor Veliparib restores autophagic flux in α‐synucleinA53T model of PD

**Figure 3:**
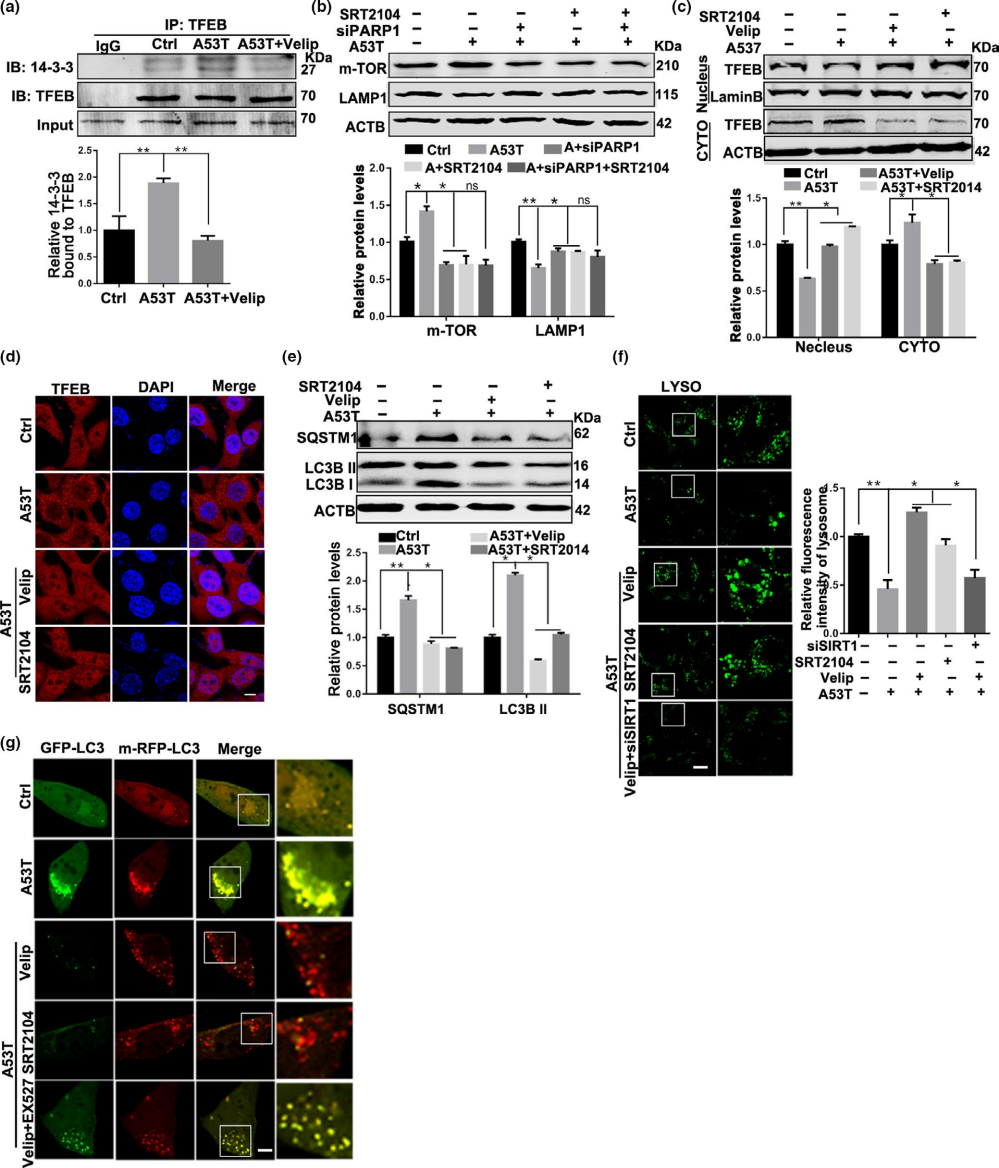
Veliparib rescues the autophagy flux via activating SIRT1 in α‐synucleinA53T model of Parkinson's disease. (a) SN4741 cells were transfected with Adenovirus‐Ctrl/A53T and further treated with Veliparib (10 μM, 12 hr). The interaction between 14‐3‐3 and TFEB was tested in TFEB Immunoprecipitates. Means ± *SEM*, *n* = 3. (b) Representative immunoblots and quantification of the levels of mTOR, LAMP1 in SN4741 cells with different treatments. Mean ± *SEM*, *n* = 3. (c) Representative immunoblots and quantification of the levels of TFEB from nuclear and cytoplasmic fractions in SN4741 cells with different treatments. (d) Immunostaining of TFEB (red) and DAPI (blue). Scale bars, 7 μm. (e) Representative immunoblots and quantification of the levels of SQSTM1, LC3B in SN4741 cells with different treatments. Mean ± *SEM*, *n* = 3. (f) Representative images and quantification of Lysosomes (green) in SN4741 cells transfected with Adenovirus‐Ctrl/A53T and further treated with Veliparib (10 μM, 12 hr), SRT2104 (10 μM, 12 hr), Veliparib plus EX527 (10 μM, 12 hr). Scale bars, 10 μm. (g) Representative images of LC3 puncta in SN4741 cells transfected with GFP‐mRFP‐LC3B plasmid. Scale bars, 5 μm (the statistical significantly was analyzed by unpaired Student's *t* test or one‐way ANOVA, *p < .05, **p < .01, and ***p < .001). TFEB, transcription factor EB

To investigate whether PARP1 over‐activation is involved in autophagy dysfunction in PD, we treated α‐synucleinA53T SN4741 cells with PARP1‐specific inhibitor Veliparib (10 μM, 12 hr) or siPARP1. Great inhibition of PAR was observed (Figure S2a). PARP1 inhibition reduced the expression of mTOR (Figure S2b) as well as the interaction between TFEB and 14‐3‐3 in α‐synucleinA53T SN4741 cells (Figure 3a). Importantly, up‐regulated nuclear accumulation of TFEB was identified (Figure S2c). These results proved that PARP1 inhibition is beneficial to TFEB nuclear localization. Next, we tested whether decreased lysosomes due to α‐synucleinA53T could be reversed by PARP1 inhibition. In fact, inhibition of PARP1increased LAMP1 expression and the number of lysosomes in α‐synucleinA53T SN4741 cells (Figure S2b,d).

To further explore the underlying effects of PARP1 inhibition on α‐synucleinA53T SN4741 cells, we detected the level of autophagy‐associated proteins. The results showed that both LC3B II and SQSTM1 reduced in responded to Veliparib treatment (Figure S2e), whereas the mRNA levels of LC3 and LAMP1 increased (Figure S2f), indicating that autophagy was effectively activated by inhibiting PARP1. Fluorescent‐tagged LC3 (GFP‐mRFP‐LC3) was used to monitor autophagy flux based on the stability of green and red fluorescent proteins in different pH (Lopez, Fleming, & Rubinsztein, 2018). Autophagy flux is enhanced when both red and yellow puncta in the cell increased, while autophagy flux is blocked only the yellow puncta increased without the increase of red puncta. Of note, results of LC3B fluorescent reporter showed Veliparib could effectively restore autophagy flux in α‐synucleinA53T SN4741 cells, as manifested in an increase in RFP‐LC3B puncta which indicated smooth fusion of autophagosomes and lysosomes (Figure S2g). Taken together, inhibition of PARP1 normalizes autophagy flux through mTOR‐TFEB pathway in PD model.

### 2.4 Veliparib rescues autophagy flux via activating SIRT1 in PD model

PARP1 and SIRT1 are both consumers of NAD^+^. It has been reported that pharmacologic inhibition of PARP1 boosts NAD^+^ content and SIRT1 activity (Bai et al., 2011). Thus, we speculated that activation of SIRT1 may be related to autophagy regulated by PARP1. After Veliparib treatment, the NAD^+^ content increased (Figure S3a). Also, the level of acetylation of PGC‐1α, as a target of SIRT1, increased in the aggregation of α‐synuclein, but decreased significantly with Veliparib treatment, which supported that the activity of SIRT1 is regulated by PARP1 (Figure S3b). As altered SIRT1 activity could negatively impact on mTOR (Guo et al., 2011), we also test whether increased SIRT1 activity can improve the autophagy ability. Results shown that SRT2104 (10 μM, 12 hr), the selectively agonist of SIRT1, decreased the expression of mTOR while increased LAMP1 in α‐synucleinA53T SN4741 cells, same as PARP1 inhibition (Figure 3b). However, there was no obvious additive effect in siPARP1 plus SRT2104 treatment (Figure 3b). Besides, the nuclear localization of TFEB increased in SRT2104 treatment (Figure 3c,d), suggesting that SIRT1 is related to mTOR‐TFEB signal. Importantly, SRT2014 diminished the expression of LC3B II and SQSTM1, testifying for enhanced autophagy flux (Figure 3e).

To evaluate the important role of SIRT1 in PARP1‐mediated autophagy dysfunction, α‐synucleinA53T SN4741 cells were treated with SIRT1 inhibitor EX527 (10 μM, 12 hr) or siSIRT1 on the basic of inhibiting PARP1. In line with the increased mTOR and reduced LAMP1 (Figure S3c), TFEB nuclear translocation was blunted to SIRT1 suppression (Figure S3d). Furthermore, SIRT1 inhibition also reduced cellular lysosomes (Figure 3f) and destroyed autophagy flux (Figure 3g). In short, the activity of autophagy elicited by PARP1 inhibition, which can be abolished by SIRT1 inhibition, indicating that SIRT1 is a key mediator for PARP1 to regulate metabolic effects.

### 2.5 Inhibiting PARP1 increases α‐synuclein degradation and reverses cell viability in PD model

**Figure 4:**
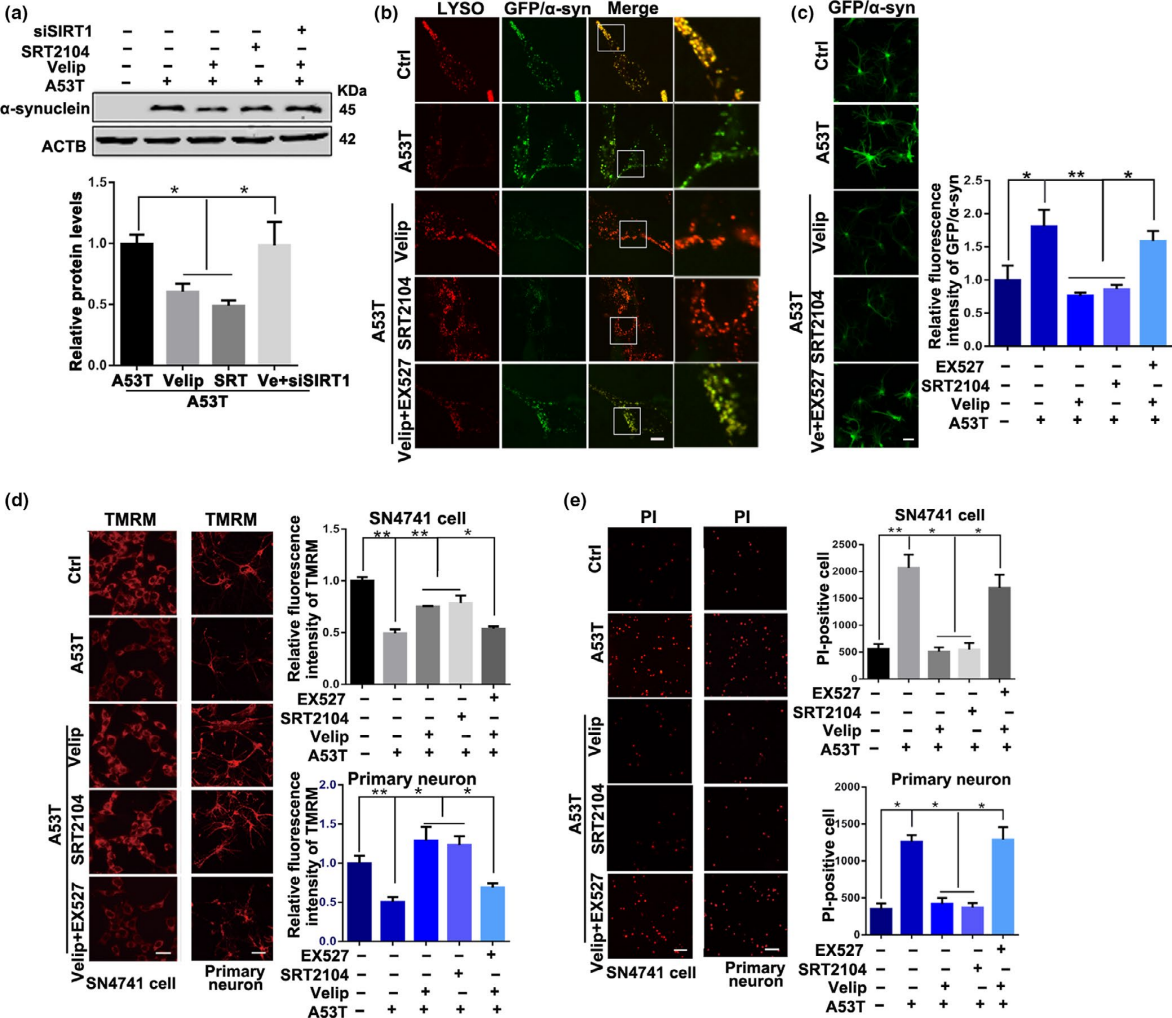
PARP1 inhibitor Veliparib treatment increases α‐synuclein degradation and reverses pathological symptoms in vitro model of Parkinson's disease. (a) Representative immunoblots and quantification of the level of α‐synuclein in SN4741 cells with different treatments. Mean ± *SEM*, *n* = 3. (b) Immunostaining of lysosomes (red) and GFP/GFP‐α‐synuclein (green) in SN4741 cell with various treatments. Scale bars, 5 μm. (c) Immunostaining of GFP/GFP‐α‐synuclein in primary cortex neurons. Scale bars, 10 μm. (d), staining of TMRM in SN4741 cell and primary cortex neurons with various treatment. Scale bars, 15 μm. (e) PI staining in SN4741 cell and primary cortex neurons Scale bars, 20 μm (the statistical significantly was analyzed by unpaired Student's *t*‐test or one‐way ANOVA, *p < .05, **p < .01, and ***p < .001). PARP1, Poly (ADP‐ribose) polymerase 1; TMRM, tetramethylrhodamine methyl ester

The above results suggested that PARP1 inhibitor Veliparib improved autophagy via SIRT1 in α‐synucleinA53T SN4741 cell. As α‐synuclein is degraded by autophagy–lysosomal pathway, we speculated that inhibition of PARP1 activity may promote α‐synuclein degradation and increase cell viability. The autophagy agonist, rapamycin (1 μM, 12 hr) and trehalose (1 μM, 12 hr), reduced the aggregation of α‐synuclein in α‐synucleinA53T SN4741 cells, supporting the degradation of α‐synuclein by autophagy (Figure S3e). As expected, Veliparib and SRT2014 treatment had a sharply reducing amount of α‐synuclein, characterized by the lessened intense band and green fluorescence in SN4741 cells and primary cortex neurons (Figure 4a–c). There is no significant decrease in the level of mRNA of α‐synuclein in α‐synucleinA53T SN4741 cells after intervening PARP1 or SIRT1 (Figure S3f). Importantly, the decreased α‐synuclein levels caused by Veliparib were inhibited by SIRT1 inhibition‐EX527, which supported that the α‐synuclein degradation depended on SIRT1. PARP1inhibition boosts SIRT1 activation, leading to an increase in mitochondrial metabolism. Veliparib and SRT2104 restored mitochondrial morphology (Figure S3g) and improved MMP (Figure 4d) in SN4741 cells and primary cortex neurons. Simultaneously, the apoptosis rate reduced significantly (Figure 4e), supporting that both inhibition of PARP1 and activation of SIRT1 had protective effects on cells survival. Of note, the protective effects of PARP1 inhibition were eliminated by inhibiting SIRT1 in PD model.

### 2.6 PARP1 regulated TFEB’s subcellular localization via affecting the interaction of TFEB‐CRM1

**Figure 5:**
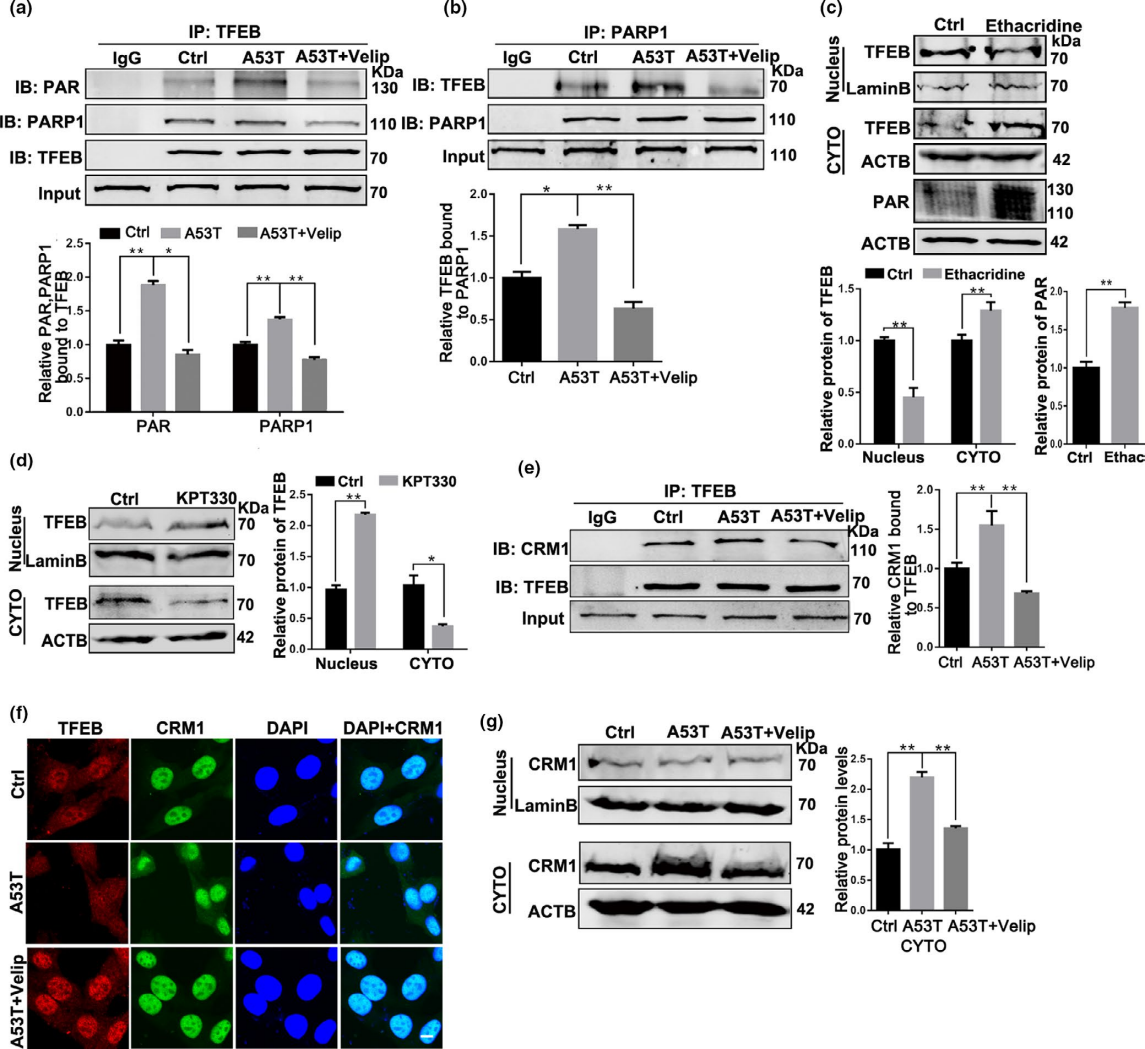
Veliparib treatment promotes nuclear localization of TFEB via reducing the interaction of TFEB‐CRM1. (a, b) SN4741 cells were transfected with Adenovirus‐Ctrl/A53T and further treated with Veliparib (10 μM, 12 hr). The levels of PAR and PARP1 interacted with TFEB were measured and analyzed in TFEB immunoprecipitates (a). The levels of TFEB interacted with PARP1 also tested and analyzed in PARP1 immuoprecipitates (b). Means ± *SEM*, *n* = 3. (c) Representative immunoblots and quantification of the levels of TFEB from nuclear fractions, cytoplasmic fractions and the levels of PAR from total lysate in SN4741 cells with Ethacridine (5 μm, 12 hr) treatments. (d) Representative immunoblots and quantification of the levels of TFEB from nuclear and cytoplasmic fraction in cells treated with KPT‐330 (5 μm, 12 hr). (e–h) SN4741 cells were transfected with Adenovirus‐Ctrl/A53T and further treated with Veliparib (10 μM, 12 hr). Then, the interaction between CRM1 and TFEB (e), Immunostaining of TFEB (red) and CRM1 (green; g), and levels of CRM1 from nuclear fractions and cytoplasmic fractions (h) were tested and analyzed in SN4741 cells. Scale bars in g, 7 μm (the statistical significantly was analyzed by unpaired Student's *t* test or one‐way ANOVA, *p < .05, **p < .01, and ***p < .001). PARP1, Poly (ADP‐ribose) polymerase 1; TFEB, transcription factor EB

Studies have confirmed that PARP1 regulates nuclear proteins’ function via PARylation (Kanai et al., 2007). Thus, we wonder whether PARP1 directly modifies TFEB by PARylation in the nucleus. To test the hypothesis, PARP1/TFEB interaction as well as TFEB PARylation were detected in SN4741 cells (Figure 5a,b). Veliparib treatment can effectively decline the increased interaction of PARP1/TFEB and TFEB PARylation caused by α‐synuclein aggregation.

To confirm whether TFEB is a new substrate of PARP1, we further examined whether PAPR1 modification affects subcellular localization of TFEB. SN4741 cells were treated with Ethacridine lactate (5 μM, 12 hr) that sharply increased the level of PAR, and the nuclear localization of TFEB decreased significantly (Figure 5c), which was consistent with the reduced levels of LAMP1 mRNA (Figure S3h), indicating that PARP1 activation is crucial for TFEB activity.

The nuclear export of TFEB depends on the combination of transporter CRM1 (Silvestrini et al., 2018). TFEB forms a nuclear export complex with CRM1 and then transits into the cytoplasm from nucleus. In fact, inhibition of CRM1 with KPT330 (10 μM, 12 hr) increased nuclear accumulation of TFEB (Figure 5d). Data showed TFEB accumulation in the nucleus following PARP1 inhibition; thus, we examined the effect of PARP1 activation on TFEB‐CRM1 interaction. Co‐IP analysis confirmed that in α‐synucleinA53T SN4741 cells, the interaction of TFEB‐CRM1 was weakened after treatment with Veliparib, resulting in the redistribution of TFEB into nucleus (Figure 5e). Interestingly, the level of CRM1 also decreased in the cytoplasm after treatment of Veliparib (Figure 5f,g). Collectively, PARP1 inhibits TFEB activity via TFEB PARylation, which depends on the interaction of TFEB‐CRM1 in SN4741 cells.

### 2.7 Suppressing PARP1 activation ameliorates pathological symptoms in α‐synuclein^A53T^‐tg mice

**Figure 6:**
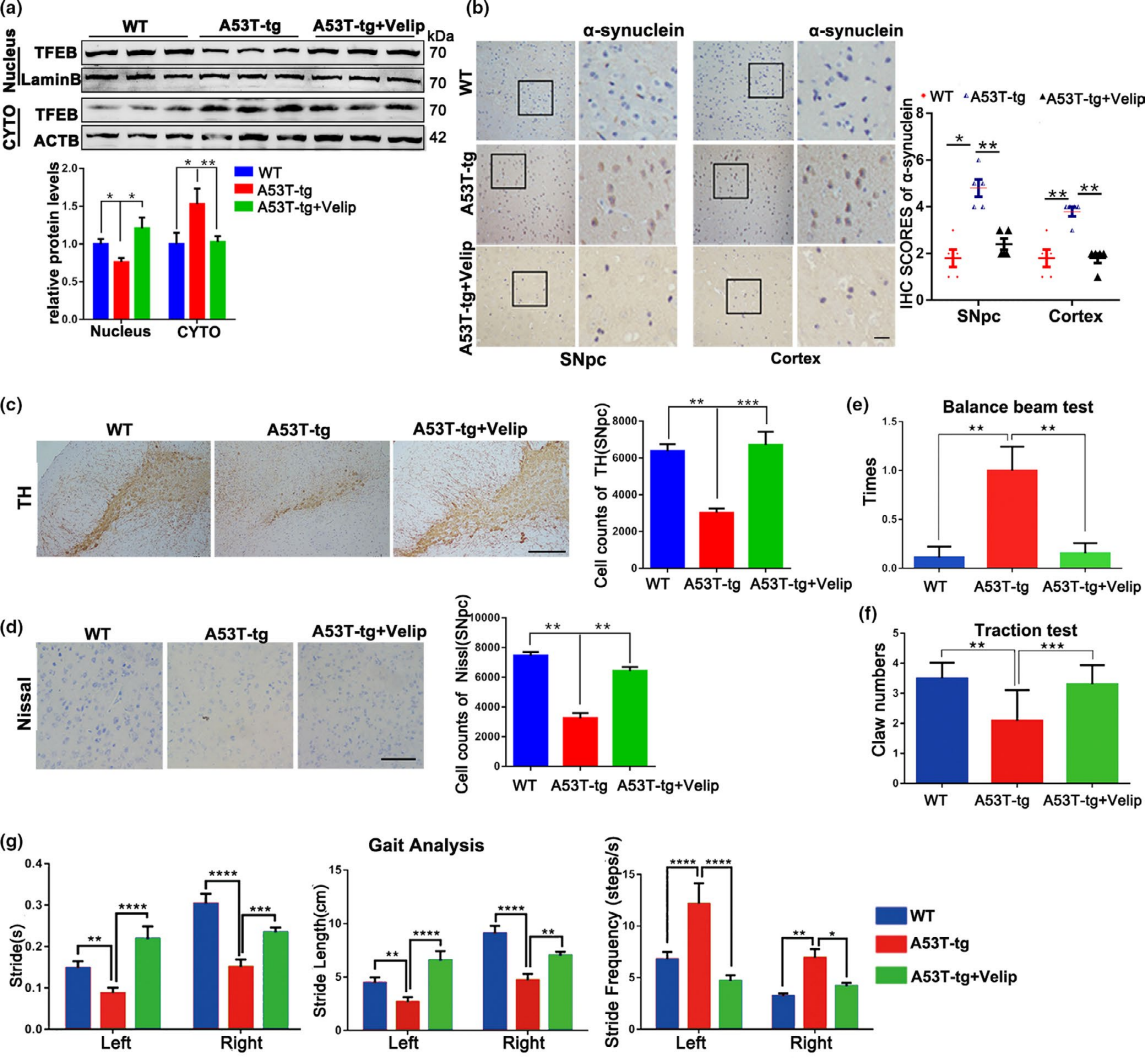
Veliparib treatment alleviates neurotoxicity and improves movement ability in α‐synucleinA53T‐tg mice. (a) Representative Immunoblots and quantification of the levels of TFEB from nuclear and cytoplasmic fractions in SNpc section of wild‐type (WT)/A53T‐tg/A53T‐tg + Veliparib mice. (b) Representative α‐synuclein staining of SNpc and Cortex tissue of WT mice, α‐synuclein^A53T^‐tg mice fed with Veliparib at 6 months or not. Statistical analysis of the scores of α‐synuclein staining shown in right. Scale bars, 200 μm. (c) Representation images of TH‐positive SNpc neurons of mice. Quantification of number in right. Scale bars, 400 μm. Means ± *SEM*, *n* = 5. (d) Representation images of Nissl‐positive SNpc neurons of mice. Quantification of number in right. Scale bars, 200 μm. Means ± *SEM*, *n* = 5. (e–g) The Balance beam test (e), Traction test (f) and Gait analysis (g) were performed in 9‐month‐old WT and α‐synuclein^A53T^‐tg mice fed with Veliparib at 6 months or not. Means ± *SEM*, *n* = 6 (the statistical significantly was analyzed by unpaired Student's *t* test or one‐way ANOVA, *p < .05, **p < .01, and ***p < .001). TFEB, transcription factor EB

To further identify the protective role of PARP1 inhibitor in vivo model of PD, 6‐month‐old α‐synuclein^A53T^‐tg mice were fed with Veliparib (50 µg/ml) in their drinking water for 3 months. Veliparib treatment vigorously increased TFEB nuclear localization (Figure 6a), associated with the reduced mTOR expression (Figure S4a) and decreased interaction of TFEB‐CRM1 (Figure S4b). In SNpc section of Veliparib‐treated α‐synuclein^A53T^‐tg mice, the activity of autophagy was also improved, as evidenced by reduced expression of LC3B II and SQSTM1, as well as increased LAMP1 (Figure S4c). Importantly, the accumulation of α‐synuclein was also attenuated in response to Veliparib detected by immunoblot and IHC (Figure S4c; Figure 6b). Because α‐synuclein is closely linked to the neurotoxicity in PD, we wonder whether motor ability in α‐synuclein^A53T^‐tg mice will be improved with inhibition of Veliparib. Supporting this premise, IHC analysis showed that Veliparib raised the number of DA neurons and Nissl's body in SNpc of α‐synuclein^A53T^‐tg mice (Figure 6c,d). Furthermore, accumulated α‐synuclein caused behavior deficits on the balanced beam test and traction test, whereas there were no obviously defects in Veliparib‐treated α‐synuclein^A53T^‐tg mice (Figure 6e,f; Movie S1 and S2). Gait analysis also showed a better performance in Veliparib fed mice (Figure 6g). Taken together, suppressing PARP1 activity normalized autophagy function, reduced neurotoxicity and recovered motor ability of α‐synuclein^A53T^‐tg mice.

## 3 DISCUSSION

This study links activated PARP1 to α‐synuclein aggregated in PD and demonstrates mechanisms that PARP1 inhibitor treatment increases the α‐synuclein degradation via up‐regulating autophagy ability. We studied the network related to autophagy‐lysosome through using PARP1 inhibitors and SIRT1 agonist in a PD model of α‐synuclein aggregation. PARP1 not only regulates the TFEB nuclear translocation through the SIRT1‐mTOR pathway, but also affects the nuclear export mediated by CRM1 through TFEB PARylation, indicating that activation of PARP1 destroys TFEB‐mediated autophagy. Combined with immunoblotting and fluorescence immunoassay, we identified that PARP1 inhibition promoted the degradation of α‐synuclein and reduced cell apoptosis. In addition, behavioral studies confirmed that the motor ability was decreased in α‐synuclein A53T transgenic mice, while improved through treating with PARP1 inhibitor that increased the number of dopaminergic neurons. These results provide new explanations for two central mechanisms of PD: DNA damage and neurodegeneration, and propose a potential treatment strategy.

Neurons rely on protein quality control to maintain the cellular homeostasis. Autophagy is a key degradation pathway for cell to keep homeostasis and plays an important role in neuronal function and survival. Emerging studies demonstrated that defects in autophagy underlie the pathogenesis of familial and idiopathic PD (Winslow & Rubinsztein, 2011). As a member of the MiT‐TFE helix‐loop‐helix leucine‐zipper (bHLH‐Zip) family of transcription factors, TFEB positively regulates autophagy and lysosome biogenesis. The activity of TFEB is modulated by protein translation modification, such as phosphorylation through mTOR, ERK, and AKT (Gao & Si, 2018; Palmieri et al., 2017), thus affecting the nuclear‐cytoplasmic shuttle of TFEB. In our study, accumulation of mutant α‐synucleinA53T led to over‐activation of PARP1, cytoplasm translocation of TFEB and autophagy dysfunction. PARP1 activation leads to cell death via inhibiting autophagy. Our data reveal the two ways of cytoplasm translocation of TFEB induced by PARP activation in PD models: (a) PARP1‐SIRT1‐mTOR pathway inhibits nuclear translocation of TFEB through phosphorylation and (b) over‐activation of PARP1 promotes TFEB PARylation, resulting in CRM1‐dependent nuclear export. PARP1 inhibitor improved PD phenotypes via up‐regulation of autophagy through increasing nuclear localization of TFEB. Importantly, the number of autophagosomes in PD increased but failed to fuse with lysosomes in PD, and PARP1 inhibitor promoted the formation of autolysosome. It is worth noting that our results demonstrate that TFEB PARylation linked to autophagy dysfunction. However, the relation between TFEB PARylation and TFEB phosphorylation involving in the modulation of TFEB requires further study.

Sirtuins is a family of proteins that catalyze deacetylation or single ADP ribosylation of target proteins using NAD^+^ as a common substrate (Imai & Guarente, 2014). One of their main characteristics is that the relatively high Km for NAD^+^, which makes NAD^+^ become the rate‐limiting substrate for their reaction. Our data showed that SIRT1 was activated after inhibiting PARP1 and promoted autophagy flux. Also, the protective effects of PARP1 inhibitor were abolished in SIRT1 knockout. PARP1 and SIRT1 bind to NAD^+^ competitively. There are reported the difference of K_M_ and Kcat/K_M_ of enzymes for NAD^+^, and the consummation of NAD^+^ by activated PARP1 is faster and more conspicuous than that of SIRT1 (Bai et al., 2011), which means that the degree of PARP1 activation determines the availability of SIRT1 by NAD^+^ limitation. Besides, SIRT1 agonist, but not the PARP1 inhibitor, can reduce DNA damage in PD models (Figure S3c). The reason for repair of DNA damage in SIRT1 agonist may be related to the deacetylation of Ku70/Ku8 which promotes the DNA repair (Jeong et al., 2007). However, we cannot give the explanation why PARP1 inhibitor have no effect on DNA repair although releasing SIRT1 activity. Hence, it is worthy to further explore the potential, complex molecular mechanism between PARP1, SIRT1, and DNA repair.

In summary, our results provided evidences that PARP1 inhibitor can be a new valuable therapy for PD. It is worth noting that PARP1 inhibitors have been approved by the Food and Drug Administration to treat several type of cancer, such as breast cancer. Thus, drug repurposing of PARP1 inhibitor can be used as a therapeutic strategy for treating PD, which reduces the cost and safety failures of the development of new drugs. Collectively, we suggest that PARP1 inhibition could be a promising, realizable intervention against PD through regulating TFEB‐mediated autophagy.

## 4 MATERIALS AND METHODS

### 4.1 Constructs, antibody, and chemicals

EGFP‐TFEB WT (38,119), mRFP‐GFP‐LC3B (84,573), EGFP‐α‐synucleinA53T (40,823), and PHM‐α‐synucleinA53T (40,825) were supplied by Addgene. GFP‐Adenovirus (AdV) and A53T‐AdV were purchased from Obio Technology. Corp. Ltd. anti‐α‐synuclein (10842‐1‐AP), anti‐PARP1 (13371‐1‐AP), anti‐TFEB (13372‐1‐AP), anti‐m‐TOR (20657‐1‐AP), anti‐LAMP1 (21997‐1‐AP), and anti‐Lamin B (12987‐1‐AP) were purchased from Proteintech; anti‐p‐m‐TOR (#5536), anti‐SIRT1 (#8469), anti‐β‐ACTB (#4970), anti‐LC3B A/B (#4211), and anti‐PGC‐1α (#2178s) were purchased from CST; anti‐TH (Santa, sc‐25269); anti‐γ‐H2A.X (Abcam, ab243906); anti‐PAR (Trevigen,4335‐MC‐100); Veliparib (Selleck, s1004); Veliparib (TargetMol; T2591); SRT2104 (Selleck, s7792); EX527(Selleck, s1541); CQ (Selleck, s8808); rapamycin (MedChenExpress, HY‐10219); and KPT‐330 (Selleck, s725).

Primers:

ACTB: F‐CATTGCTGACAGGATGCAGAAGG‐, R‐TGCTGGAAGGTGGACAGTGAGG‐

LC3B: F‐GGACCTGCTGCTTCTCTAA‐, R‐ACTGCTGAGTGAAAGGGTGT‐

LAMP1: F‐AGCCCTGGAATTGCAGTTTG‐, R‐CACTGTCCACCTTGAAAGCC‐

α‐synucleinA53T‐tg: F‐TGTAGGCTCCAAAACCAAGG‐, R‐TGTCAGGATCCACAGGCATA‐

siPARP1: F‐GCAGCGAGUAGUAUUCCCAAdTdT‐, R‐UUGGGAAUACUCUCGCUGCdTdT‐;

siSIRT1: F‐ACGAUGACAGAACGUCACAdTdT‐, R‐UGUGAGUUCUGUCAUCGUdTdT‐

#### 4.1.1 Animal

The α‐synuclein^A53T^‐tg mice were obtained from the Model Animal Research Center of Nanjing University. The mice were kept in separate captivity in the Experimental Animal Center of Southern Medical University, Grade SPF, light: dark (12:12 hr) circulation, providing food and water.

#### 4.1.2 Balance beam test

Mice were placed on a narrow, smooth beam (20 mm/12 mm width, 80 cm long) suspended at a height of 20 cm. Impelling the mice moved from one end to the other, recording the number of missteps (foot slips) during the trip. Before the formal test, mice were trained several times which promoted mice to familiarize themselves with trip.

#### 4.1.3 Traction test

The mouse grasped the rope with forepaws to keep it suspended in air. Within 5 s, the number of claws held on the rope was recorded.

#### 4.1.4 Gait analysis

Mice were placed on a transparent board and their movement in 3 s was recorded with camera under the board. The gait date of mice were collected and analyzed from different aspects.

#### 4.1.5 Immunohistochemistry

Mice were perfused with physiological saline, and the brains were taken and then fixed with paraformaldehyde. The brain tissues were embedded in paraffin, and the serial sections (40 μm thick) were stained for IHC. Primary antibodies and working dilution were worked according to the manufacturer's instructions. In this study, striatum and substantia nigra sections were staining for TH and Nissl‐positive. Brain tissues were staining for mTOR, α‐synuclein, and PARP1. Both TH and Nissl‐positive DA neurons were counted by ImageJ analysis system. For the quantification, the intensity and the percentage of positive cells and staining intensity were used to calculate the expression of level of proteins. IHC images were taken under an optical microscope. Each group collected information from 10 pictures for statistical analysis.

#### 4.1.6 Primary neuronal culture

Cortical neurons cultures were prepared from suckling mice <1 day old. Dissociated cells were seeded into plastic dishes (1,500,000 cells) coated with poly‐lysine. Neuronal were maintained in DMEM/F12 supplemented with 10% fetal bovine serum (FBS) at 37℃ with 5% CO_2_ for 2 hr, and then, the medium was replaced with B‐27 Plus Neuronal Culture System supplemented with 1% L‐glutamine and 100 U/ml penicillin‐streptomycin and incubated at 37℃ with 5% CO_2_. The neurons culture media was half‐replaced with fresh medium every 3 days. On day 9, neurons were transfected with Adenovirus‐Ctrl/A53T and, on day later, adding inhibitor or agonist.

#### 4.1.7 Cell culture, transfection, and treatment

SN4741 cell were cultured with DMEM medium containing 10% FBS, 1% glucose, 100 U/ml penicillin‐streptomycin, and 1% L‐glutamine at 33℃ under 5% CO_2_. After 12 hr of seeded, cells were transfected with A53T AdV. If necessary, siRNA was transfected simultaneously. 24 hr later, drugs were added to the culture medium according to dose and time used in cells. Veliparib (10 μM), SRT2104 (10 μM), EX527 (10 μM), KPT‐330 (5 μM), and Rapamycin (20 μM) were applied to cell for 12 hr. CQ (5 μM) was applied to cell for 4 hr. The dose and time of drugs treatment in primary neuron were the same as those of SN4741 cell.

#### 4.1.8 Western blot and co‐immunoprecipitation

SNpc and cortex samples for Western blotting were prepared by tissue homogenization method. SN4741 cell and primary neuron were collected in RIPA buffers (20 mM Tris‐HCl, pH 7.4, 150 mM NaCl, and 1% Triton X‐100) containing protease inhibitor cocktail (Roche). Samples were centrifuged at 14,000 × g for 15 min at 4℃ and then quantitatively analyzed with the BCA assay. The cytosolic and nuclear fractions were collected using nuclear exaction kit (KGF1100; KeyGen). Samples were loaded on 12% SDS‐polyacrylamide gels for separation and then transferred onto PVDF membranes (Millipore). The membranes was sealed with 5% BSA at room temperature for 1 hr and probed overnight with primary antibody at 4℃. The stripe is visualized by Odyssey Infrared Imaging System. For co‐immunoprecipitation, sample were collected and detected same as immunoblot. Then, same amount of protein were incubation with 5 μg of the corresponding antibody overnight at 4℃, followed by incubation with 50 μl agarose beads for 2 hr at 4℃. The IP complexes were washed with RIPA buffer for three times. After adding 2× SDS loading buffer, the sample was denatured by boiling for 5 min.

#### 4.1.9 Immunofluorescence

Cells were seeded to confocal petri dish and cultured for 2 days. After washed with 1× PBS for three times, cells were fixed in 4% paraformaldehyde for 15 min, incubated with frozen methanol at −20°C for 10 min, and 30 min was blocked with 5% BSA at room temperature. Then, primary antibody (1:100) in 5% BSA covered cells overnight at 4℃, and then, cells incubated Alexa Flour secondary antibody (goat‐anti‐mice 488 and goat‐anti‐rabbit 594 antibodies, Thermo Fisher, 1:100) for 1 hr at room temperature. The fluorescent images were acquired by Laser Scanning Confocal Microscopy (Olympus FV1000). Each group collected information from 10 pictures or 25 cells for statistical analysis.

#### 4.1.10 Fluorescent staining of lysosome and mitochondria

Lysosomal staining and mitochondrial staining were carried as the manufacturer's instructions. SN4741 cells were seeded into confocal petri dish and cultured for 2 days. Cells were washed once with 1× PBS, and 23 min and 20 min were labeled with 0.01% LysoTracker Green/Red (Life Technologies, L7526/L7528) and 10  nM Mito Tracker Red (Life Technologies, M22425), respectively, in cell culture medium. Images were captured by Laser Scanning Confocal Microscopy. Each group collected information from 10 pictures or 25 cells for statistical analysis.

#### 4.1.11 Apoptosis and cell death

Cell apoptosis of SN4741 cell was measured using a Cell Apoptosis Kit (Dojindo) according to the manufacturer's instructions. The number of cell apoptosis was counted by flow cytometry, and images of apoptosis were taken by Laser Scanning Confocal Microscopy. Cell death of primary neuron was detected by staining with PI (1 μg/ml; KeyGEN) according to the manufacturer's instructions. Images were captured by fluorescence microscope and analyzed by ImageJ analysis software. Data collected from flow cytometry were repeated at three times. Count of PI‐positively was collected from 10 pictures each group.

#### 4.1.12 Comet assay

SN4741 cells were seeded into plastic dishes and grown for 2 days. The cells were washed with ice‐cold 1× PBS, harvested, and suspended in PBS at 1 × 10^6^ cells/ml. After mixed with 100 μl of 0.5% low melting point agarose in PBS at 37℃, 30 μl of the cell‐agarose mixture was immediately placed on the Comet side at 4℃ for 30 min in the dark. Slides were lysed in lysis buffer and immersed in alkaline unwinding solution overnight at 4℃. Next day, electrophoresis at 20 V, 300 mA for 20 min, the slides were rinsed three times with neutralizing buffer (Tris base 48.5 g, ddH_2_O 1,000 ml, pH = 7.5). Subsequently, the slides were fixed with 70% ethanol for 5 min and then stained with EB application liquid (5 μg/ml) for 5–10 min. Images were captured using a fluorescence microscope.

#### 4.1.13 NAD^+^ assay and ATP assay

The content of NAD^+^ and ATP of SN4741 cells was measured using a NAD^+^/NADH Assay Kit with WST‐8 (Beyotime, S0175) and Enhanced ATP Assay Kit (Beyotime, S0027), and was measured by spectrometer and luminometer, respectively. Carry out related operations according to the manufacturer's instructions.

#### 4.1.14 Mitochondrial membrane potential

The MMP in cells was assessed using tetramethylrhodamine methyl ester (TMRM; Thermo Fisher, T668). Cells were washed with 1× PBS and then incubated with TMRM (0.1 μM) in medium for 20 min in the dark. The intensity of fluorescence was monitored using fluorescence microscope.

### 4.2 Statistical analysis

All data are represented as mean ± *SEM*. There are at least three separate experiments. Use GraphPad Prism 6 software to analyze the data. Student's *t* test and ANOVA were used to compare the differences between the two methods and multiple methods. The assessment of *p* < .05 is considered to be meaningful.

## CONFLICT OF INTEREST

The authors have no conflicting financial interest.

## AUTHORS’ CONTRIBUTIONS

K‐M M and J‐L C designed and conceptualized the research, did the experimental work, and analyzed data. H‐L Y, H‐H L, Y‐Y R, X‐W, Y‐W, and K‐M M provided technical assistance. W‐J L, K‐M M, and J‐L C wrote the manuscript. F Z and W‐J L is the senior author who designed the project.

## Supporting information

Supplementary Material

Movie S1

Movie S2

## Data Availability

The data that support the findings of this study are available from the corresponding author upon reasonable request.
