# Supplementary material for: Poly (ADP‐ribose) polymerase 1 inhibition prevents neurodegeneration and promotes α‐synuclein degradation via transcription factor EB‐dependent autophagy in mutant α‐synucleinA53T model of Parkinson's disease
Source: Aging Cell. 2020 May 31;19(6):e13163. doi: 10.1111/acel.13163 (PMC7294777; doi:10.1111/acel.13163)
Supplement: Supplementary file 1 — Supplementary Material [file ACEL-19-e13163-s001.zip › acel13163-sup-0005-Supinfo.docx]

**Graphical Abstract**

α-synuclein aggregated induced the DNA damaged and PARP1 activation, which down-regulated TFEB nuclear translocation via TFEB PARylation or TFEB phosphorylation regulated by PAR and SIRT1-m-TOR pathways, separately. Inhibition of PARP1 promotes TFEB-mediated autophagy and then increases the degradation of α-synuclein, providing a potential therapeutic strategy of Parkinson’s disease.

**Figure legends (supplement)**

**Fig. S1**

**(**a, b) Representative Immunoblots and quantification of the levels of α-synuclein in α-synucleinA53T-tg or wild type mice (a) and in SN4741 cells (b). Mean ± SEM, n=3. (c) Tail DNA from α-synucleinA53T-tg or wild type mice were isolated and detected by PCR. (d) The SN4741 cells transfected with Adenovirus-A53T or not were tested by comet assay. Scale bars, 10μm. (e) The protein levels of LC3B II and SQSTM1 in SN4741 cell that overexpressed of TFEB or treated with Rapamycin (20 μM, 12h) were detected by Immunoblot. Mean ± SEM, n=3. (The statistical significantly was analyzed by unpaired Student’s *t*-test, **p* <0.05, ***p* <0.01 and ****p* <0.001)

**Fig. S2**

(a, b) Representative Immunoblots and quantification of the levels of PAR in SN4741 cells with PARP1 inhibition. Mean ± SEM, n=3. (b) Representative Immunoblots and quantification of the levels of m-TOR and LAMP1 in SN4741 cells with different treatment. (c)Subcellular localization of TFEB was analyzed by confocal fluorescence microscopy with Immunofluorescence. Scale bars, 5μm. (d) The lysosomes were stained lysosome tracker (green) in SN4741 cells with different treatment. And the relative fluorescence intensity of lysosomes was statistically analyzed. Scale bars, 10μm. (e) Representative Immunoblots and quantification of the levels of SQSTM1 and LC3B in SN4741 cells with different treatment. (f) The mRNA level of LC3B and LAMP1 in SN4741 cells were detected by PCR. (g) Representative images of LC3 puncta in SN4741 cells transfected with GFP-mRFP-LC3B plasmid. Scale bars, 5μm. (The statistical significantly was analyzed by unpaired Student’s *t*-test, **p* <0.05, ***p* <0.01 and ****p* <0.001)

**Fig. S3**

The levels of NAD^+^ in SN4741 cells were measured by spectrophotometer. (a) NAD^+^ levels in SN4741 cells was measured by spectrophotometer. (b) Cell lysates from SN4741 cells were used for IP, using anti-PGC-1α antibody. Veliparib reduced the Ac-Lys/PGC-1α interaction increased by Adenovirus-A53T. Relative proteins level were statistically analyzed. (c) Representative Immunoblots and quantification of the levels of m-TOR, LAMP1 and γ -H2A.X in SN4741 cells with different treatment. Mean ± SEM, n=3. (d) Subcellular localization of TFEB in SN4741 cells was detected by confocal microscopy with Immunofluorescence. Scale bars, 7μm. (e)Representative Immunoblots and quantification of the levels of α-synuclein in SN4741 cells with various treatments. (f) The mRNA level of α-synuclein in SN4741 cells were detected by PCR. (g) Representative images of mitochondrial tracker (red) staining from SN4741 cells with different treatment, and quantification of mean branch length of mitochondrial fragment. Scale bars, 5μm. (h)The mRNA level of LAMP1 in SN4741 cells treated with Ethacridine (5μM, 12h) were detected by PCR. (The statistical significantly was analyzed by unpaired Student’s *t*-test or One-way ANOVA, **p* <0.05, ***p* <0.01 and ****p* <0.001).

**Fig. S4**

(a) Representative m-TOR staining of SNpc tissue of WT mice, α-synuclein^A53T^-tg mice fed with Veliparib at 6 months or not. Statistical analysis of the scores of m-TOR staining shown in right. Scale bars, 200μm. (b) The levels of CRM1 interacted with TFEB were measured and analyzed in TFEB Immunoprecipitates. Mean ± SEM, n=3. (c) Representative Immunoblots and quantification of the levels of LAMP1, TH, SQSTM1, α-synuclein and LC3B from SNpc and Cortex tissue in mice with different treatment. Mean ± SEM, n=3. (The statistical significantly was analyzed by unpaired Student’s *t*-test or One-way ANOVA, **p* <0.05, ***p* <0.01 and ****p* <0.001).

**Movie 1** The Balance Beam Test were used to examine the motor ability of WT, α-synuclein A53T-tg, or α-synuclein A53T-tg mice fed with Veliparib. α-synuclein A53T-tg mice showed an obvious missteps (foot slips).

**Movie 2** The Traction Test was performed in WT, α-synuclein A53T-tg, or α-synuclein A53T-tg mice fed with Veliparib.
