# Supplementary figures and images for: Poly (ADP‐ribose) polymerase 1 inhibition prevents neurodegeneration and promotes α‐synuclein degradation via transcription factor EB‐dependent autophagy in mutant α‐synucleinA53T model of Parkinson's disease
Source: Aging Cell. 2020 May 31;19(6):e13163. doi: 10.1111/acel.13163 (PMC7294777; doi:10.1111/acel.13163)

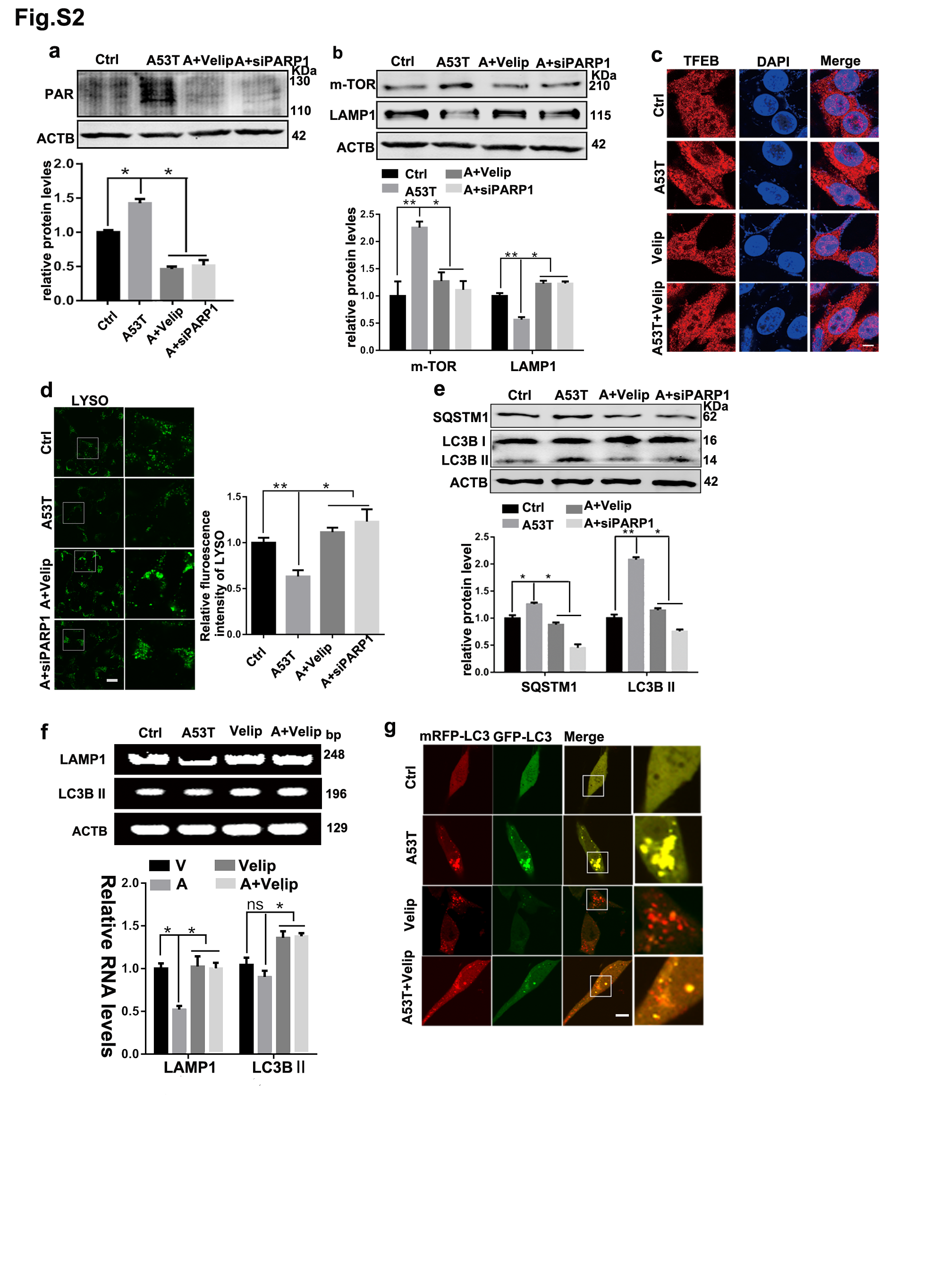

Supplement: Supplementary file 1 — Supplementary Material [file ACEL-19-e13163-s001.zip › acel13163-sup-0002-FigS2.tif]

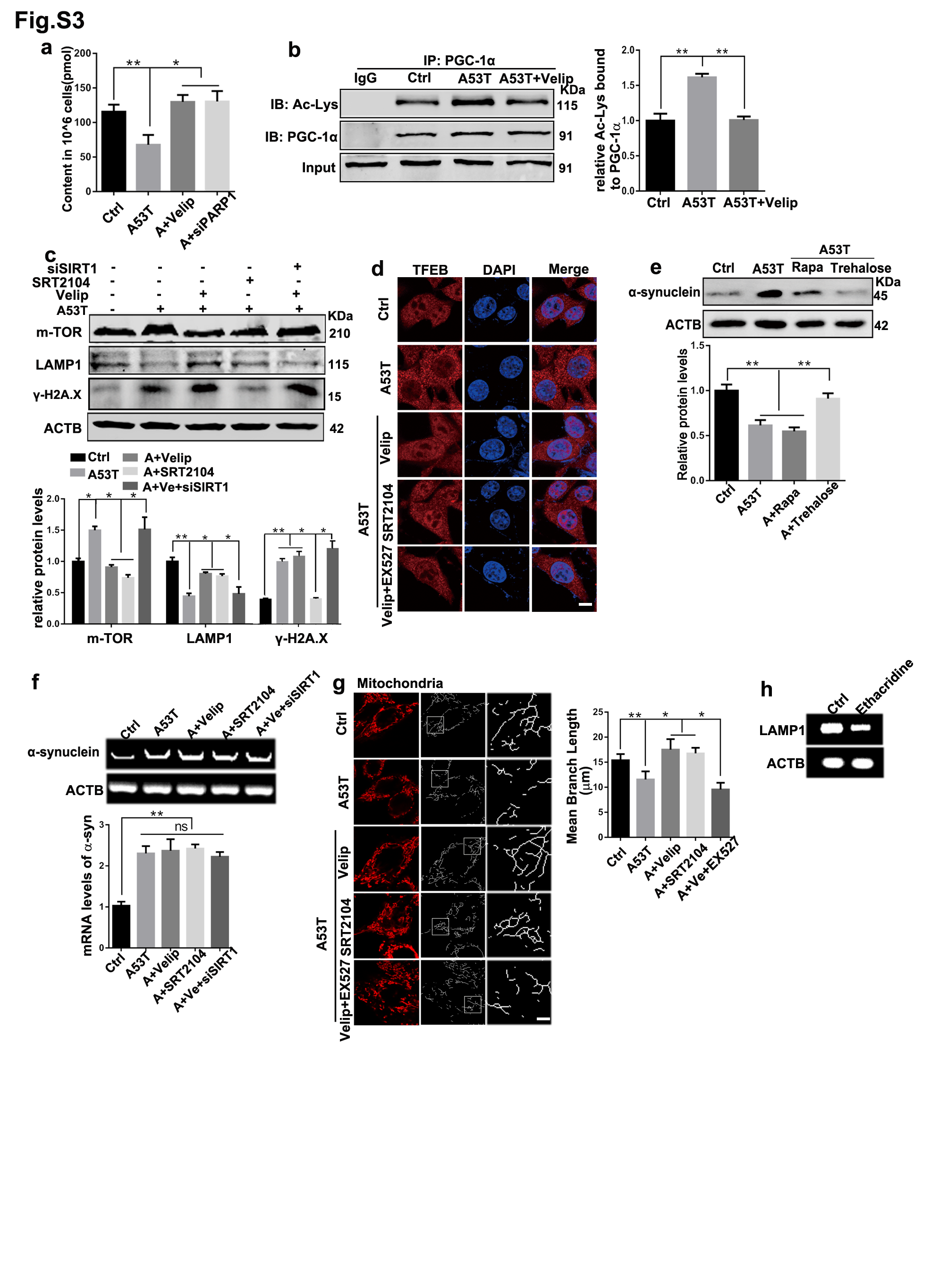

Supplement: Supplementary file 1 — Supplementary Material [file ACEL-19-e13163-s001.zip › acel13163-sup-0003-FigS3.tif]

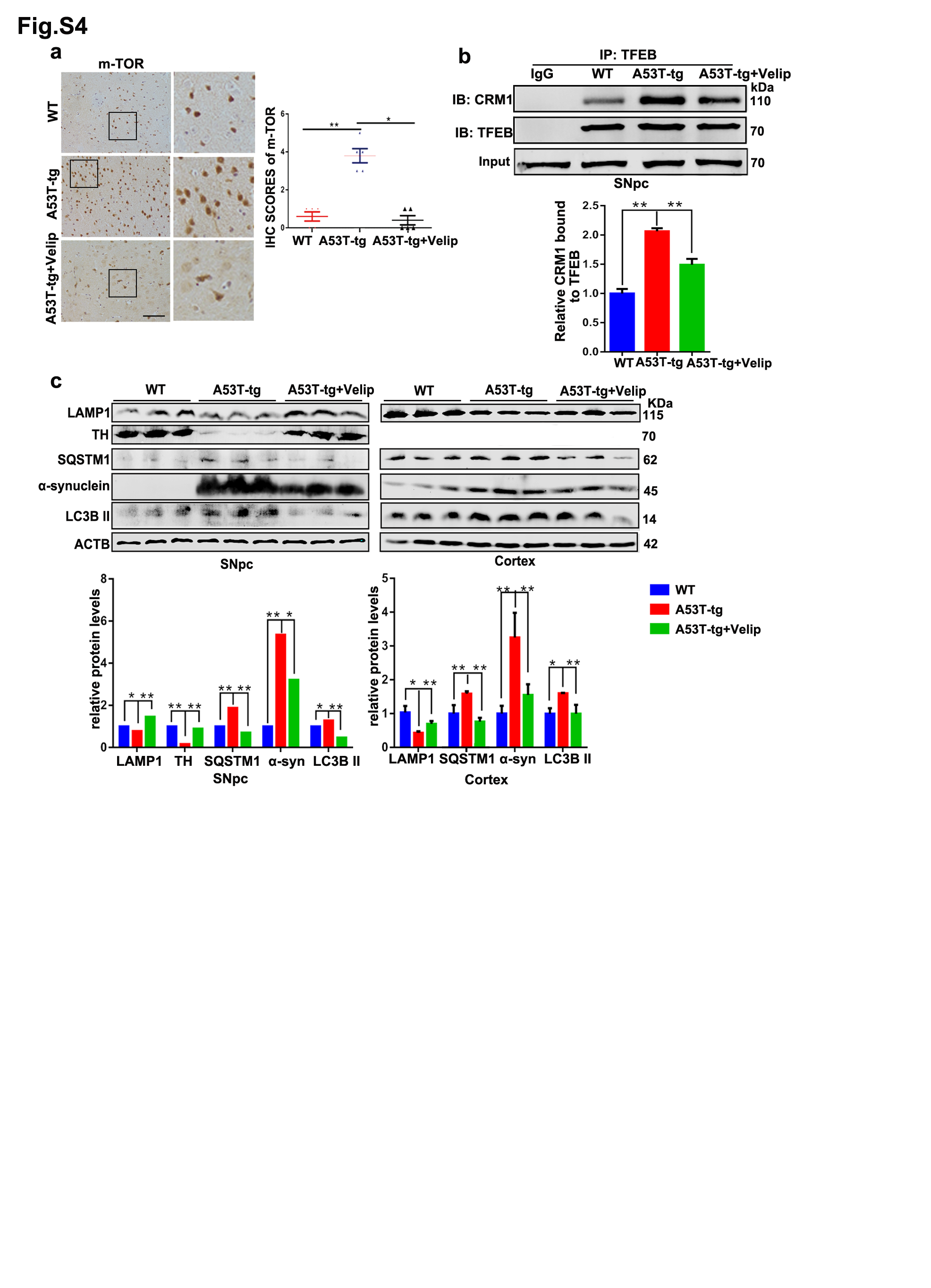

Supplement: Supplementary file 1 — Supplementary Material [file ACEL-19-e13163-s001.zip › acel13163-sup-0004-FigS4.tif]

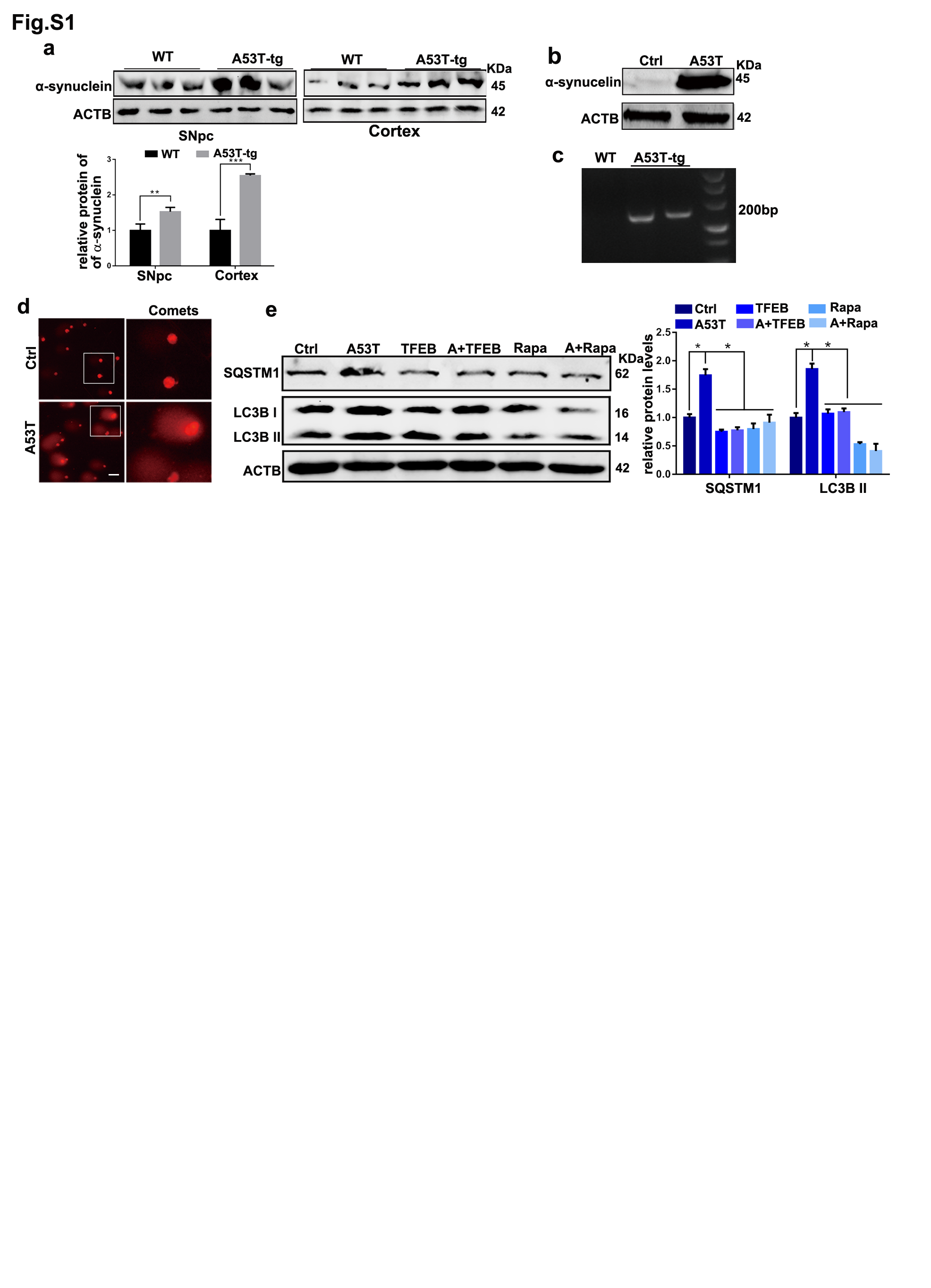

Supplement: Supplementary file 1 — Supplementary Material [file ACEL-19-e13163-s001.zip › acel13163-sup-0001-FigS1.tif]
